# Assessment of molecular markers of anti-malarial drug resistance among children participating in a therapeutic efficacy study in western Kenya

**DOI:** 10.1186/s12936-020-03358-7

**Published:** 2020-08-14

**Authors:** Winnie Chebore, Zhiyong Zhou, Nelli Westercamp, Kephas Otieno, Ya Ping Shi, Sheila B. Sergent, Kelsey Anne Rondini, Samaly Souza Svigel, Benard Guyah, Venkatachalam Udhayakumar, Eric S. Halsey, Aaron M. Samuels, Simon Kariuki

**Affiliations:** 1 Kenya Medical Research Institute, Centre for Global Health Research, P.O. Box 1578, Kisumu, Kenya; 2 Maseno University, Kisumu, Kenya; 3 Centers for Disease Control and Prevention, Malaria Branch, Atlanta, GA USA; 4 Rollins School of Public Health, Emory University, Atlanta, GA USA; 5 U.S. President’s Malaria Initiative, Atlanta, GA USA; 6 Centers for Disease Control and Prevention, Kisumu, Kenya

**Keywords:** *Plasmodium falciparum*, Anti-malarial drug resistance, *Pfk13*, *Pfmdr1*, *Pfcrt*, *Pfpm2*

## Abstract

**Background:**

Anti-malarial drug resistance remains a major threat to global malaria control efforts. In Africa, *Plasmodium falciparum* remains susceptible to artemisinin-based combination therapy (ACT), but the emergence of resistant parasites in multiple countries in Southeast Asia and concerns over emergence and/or spread of resistant parasites in Africa warrants continuous monitoring. The World Health Organization recommends that surveillance for molecular markers of resistance be included within therapeutic efficacy studies (TES). The current study assessed molecular markers associated with resistance to Artemether−lumefantrine (AL) and Dihydroartemisinin−piperaquine (DP) from samples collected from children aged 6–59 months enrolled in a TES conducted in Siaya County, western Kenya from 2016 to 2017.

**Methods:**

Three hundred and twenty-three samples collected pre-treatment (day-0) and 110 samples collected at the day of recurrent parasitaemia (up to day 42) were tested for the presence of drug resistance markers in the *Pfk13* propeller domain, and the *Pfmdr1* and *Pfcrt* genes by Sanger sequencing. Additionally, the *Pfpm2* gene copy number was assessed by real-time polymerase chain reaction.

**Results:**

No mutations previously associated with artemisinin resistance were detected in the *Pfk13* propeller region. However, other non-synonymous mutations in the *Pfk13* propeller region were detected. The most common mutation found on day-0 and at day of recurrence in the *Pfmdr1* multidrug resistance marker was at codon 184**F**. Very few mutations were found in the *Pfcrt* marker (< 5%). Within the DP arm, all recrudescent cases (8 sample pairs) that were tested for *Pfpm2* gene copy number had a single gene copy. None of the associations between observed mutations and treatment outcomes were statistically significant.

**Conclusion:**

The results indicate absence of *Pfk13* mutations associated with parasite resistance to artemisinin in this area and a very high proportion of wild-type parasites for *Pfcrt*. Although the frequency of *Pfmdr1* 184**F** mutations was high in these samples, the association with treatment failure did not reach statistical significance. As the spread of artemisinin-resistant parasites remains a possibility, continued monitoring for molecular markers of ACT resistance is needed to complement clinical data to inform treatment policy in Kenya and other malaria-endemic regions.

## Background

Malaria caused an estimated 228 million new infections and 405,000 deaths globally in 2018 [1]. In Kenya, there were over 15 million suspected and 1.5 million confirmed cases of malaria in 2018 [1], with the burden highest in areas around Lake Victoria and the coastal region [2]. Artemisinin-based combination therapy (ACT) is recommended by the Kenya Ministry of Health as first- and second-line treatments for uncomplicated malaria, and available data suggest that they remain highly effective in Kenya and throughout sub-Saharan Africa [3, 4]. However, the emergence and spread of *Plasmodium falciparum* resistance to artemisinin and partner drugs presents one of the greatest challenges to global malaria control and elimination efforts and warrants continuous surveillance of resistance in malaria endemic areas.

The World Health Organization (WHO) recommends routine surveillance for early detection of resistance or emergence of resistant parasites [5]. While in vitro, ex vivo drug testing and in vivo therapeutic efficacy studies (TES) are important for assessing the effectiveness of anti-malarial drugs [6], molecular surveillance using genetic markers associated with resistance provides a valuable tool for detecting and tracking resistance as well as providing an in-depth understanding of the development and spread of resistance. Earlier studies have shown that parasite resistance to anti-malarial drugs is often associated with single nucleotide polymorphisms (SNPs) or amplifications of the genes coding for drug target proteins or transporters [7]. For example, SNPs at codons 86, 184, and 1246 in the *P. falciparum* multidrug resistance 1 gene (*Pfmdr1*) have been implicated to confer parasite resistance to multiple anti-malarial drugs, with individual polymorphisms leading to opposite effects on different drugs [8]. Mutations at *Pfmdr1* 86**Y** and 1246**Y** have been linked to decreased sensitivity to chloroquine and amodiaquine, but increased sensitivity to lumefantrine, mefloquine and artemisinin [9–13], while mutations at 184**F** have been associated with reduced susceptibility to lumefantrine [14, 15]. Likewise, mutations at codons 72 to 76 of the *P. falciparum* chloroquine resistance transporter gene (*Pfcrt*) have been associated with resistance to chloroquine and amodiaquine [16, 17], with the 76**T** point mutation being the most predictive of chloroquine resistance. Amplification of the *plasmepsin2* gene (*Pfpm2*) has been recently associated with piperaquine resistance [18]. Additionally, polymorphisms in the *P. falciparum* kelch 13 (*Pfk13*) propeller region have been causally linked to artemisinin resistance in Southeast Asia [19, 20]  and serve as valuable molecular markers for tracking and monitoring artemisinin-resistant parasites.

Clinical trials performed in Africa have provided evidence for the selection of particular *Pfmdr1* alleles in patients with newly acquired or recurrent *P. falciparum* infections within 28 or 42 days after ACT treatment [21–25]. Additionally, genetic mutations in the *Pfk13* propeller region have been associated with delayed parasite clearance, which has been shown to lead to more treatment failures after ACT treatment [26]. While previous studies performed in western Kenya have compared the prevalence of *Pfmdr1* and *Pfk13* molecular markers before and after ACT was introduced [27–29] and assessed their association with parasite clearance rates [27], there is limited information on the association between these markers and treatment outcomes. Understanding the relationship between molecular markers, parasite resistance and treatment failure is important for evidence-based decision making on anti-malarial drug policies.

The main objective of this study was to assess the frequency of molecular markers associated with resistance to anti-malarial drugs from parasite isolates collected during a TES conducted in Siaya, western Kenya in 2016–2017. This current manuscript reports the frequencies of SNPs in genes associated with resistance or tolerance to anti-malarial drugs including, *Pfcrt* for chloroquine resistance [30], *Pfmdr1* for lumefantrine tolerance [31], *Pfk13* for artemisinin resistance [19], and amplification of *Pfpm2* for piperaquine resistance [18]. The association between these mutations with TES treatment outcome was also examined. Another manuscript in process details the efficacy and clinical endpoints [32].

## Methods

### Study area and population

The main TES, through which the samples were collected, was conducted from 2016 to 2017 as a routine surveillance activity to monitor the efficacy of Artemether–lumefantrine (AL) and Dihydroartemisinin−piperaquine (DP) for treatment of uncomplicated falciparum malaria in children aged 6–59 months in Siaya County, western Kenya, using the standardized WHO 2009 drug efficacy study protocol [33]. The study area has been described in detail [34, 35]. Briefly, it is an area of perennially high malaria transmission with seasonal peaks in June-July and November–December, following the long and short rains, respectively. Malaria prevalence by blood smear microscopy in children < 5 years of age was estimated to be 39.0% in July, 2015, and only 52.8% of those positive reported fever in the previous 2 weeks [35]. AL was scaled up as the first-line and DP as the second-line anti-malarial in 2006 and 2010, respectively. Therapeutic efficacy studies evaluating these anti-malarial have been conducted in this area in 2005–2011 [4, 36–39], and reported the drugs to be efficacious.

Children were enrolled at three health facilities: Siaya County Referral Hospital, and Mulaha and Bar Agulu dispensaries. A total of 340 participants (166 participants in the AL and 174 in the DP arm) were randomized to receive a standard weight-based regimen of AL or DP and followed for 42 days. A portion of all available dried blood spot (DBS) samples collected pre-treatment (day-0) and on the day of recurrent parasitaemia (either days 7, 14, 21, 28, 35, 42 or any smear-positive sick visit) after treatment with AL or DP were used in this study. Of the 340 study participants, 111 participants returned with a recurrent malaria infection. A total of 323 day-0 and 110 recurrent infections samples were available for analysis. Seventeen (5%) of the day-0 and 1 (0.9%) of the recurrent infection samples were either missing or the DBSs were not collected. The available samples were analysed for drug resistance markers of the *Pfk13* propeller domain, *Pfmdr1* and *Pfcrt* genes. The 110 samples from recurrent infections were genotyped to differentiate between re-infection and recrudescence. Recrudescent samples from the DP arm were analysed for amplification of *Pfpm2*.

### DNA extraction and storage

Genomic DNA was extracted from the dried blood samples using the QIAamp^®^ DNA mini kit (QIAGEN, Inc., Germany), according to the manufacturer’s instructions (DBS protocol). The extracted DNA samples were kept frozen at − 20 °C until use.

### Genotyping to characterize recurrent infections

To distinguish between re-infection and recrudescence, pre- and post-treatment samples from participants with recurrent infections were genotyped by polymerase chain reaction (PCR) based on merozoite surface proteins 1 and 2 (*msp1* and *msp2*) and glutamate-rich protein (*glurp*), as per WHO recommended genotyping procedures [40]. A sequential algorithm starting with *msp2* followed by *glurp* and finally *msp1* was used. The PCR products were run on agarose gels (3% for *msp1* and 2% for *msp2* and *glurp* alleles) pre-stained with ethidium bromide, after which the gels were observed under UV Trans illuminator at 312 nm to visualize the bands. Enumeration of DNA bands and their molecular size analysis was carried out using Lab Works software, against 100 bp standard molecular marker (New England Biolabs Ltd., Ontario, Canada). Recrudescence was defined as at least one identical allele (< 20 bp) for each of the three markers (*msp2, glurp*, and *msp1*) in the pre- and post-treatment samples [40].

### *Pfpm2* copy number amplification

*Pfpm2* copy number amplification was performed using the Agilent real-time PCR machine (Stratagene MX3005P; Agilent Technologies, La Jolla, USA) following a recently published method [41]. All samples were tested in triplicate. Two positive controls with one copy and three copies were used in this assay. The *Pfpm2* copy number was determined by the 2-ΔΔCT method (ΔCT = CT *Pfpm2* − CT *P. falciparum* β -tubulin gene; CT, threshold cycle) using the 3D7 *P. falciparum* strain, known to have a single copy of the *Pfmd2* gene, as a calibrator.

### Gene amplification of the *Pfk13* propeller domain, *Pfmdr1,* and *Pfcrt* genes

Gene amplification of the *Pfk13* propeller domain, *Pfmdr1* and *Pfcrt* genes was carried out in a 96-well format GeneAmp PCR system 9700 (Applied Biosystems, Foster City, USA). For *Pfk13*, the fragment of the gene that was amplified contained the six codons that have been validated to correlate with artemisinin resistance [5]. These codons are: 458 (N458**Y**), 493 (Y493**H**), 539 (R539**T**), 543 (I543**T**), 561 (R561**H**), and 580 (C580**Y**). Primary and nested PCR for *Pfk13* propeller region amplification was performed using 50–250 ng of DNA sample in a 25-µL PCR mix containing 0.5 µM of each primer, 0.2 µM dNTPs, 1.0 U HF Phusion DNA Polymerase and 1 × Phusion HF buffer following a previously published protocol [42]. The primary and nested *Pfmdr1* gene amplification (codons 86–184 and 1034–1246) was performed using 50–250 ng of DNA sample in a 25-µL PCR mix containing 0.5 µM of each primer and 1 × Promega Master Mix following previously published protocols [42–44]. The primary and nested PCR amplification for *Pfcrt* gene (codons 72–76) was performed using 50–250 ng of DNA sample in a 25-µL PCR mix containing a final concentration of 0.25 µM of each primer and 1 × Promega Master Mix as per a previously published protocol [45].

### Sequencing of the *Pfk13* propeller domain, *Pfmdr1* and *Pfcrt* genes

Sanger sequencing by capillary electrophoresis for the *Pfk13* propeller region, *Pfmdr1* and *Pfcrt* genes was carried out in ABI 3130XL DNA sequencer (Applied Biosystems, Foster City, USA) as previously described [44]. Geneious R10 software (Biomatters, San Francisco, USA) was used to identify specific SNPs by assembling the sequences with annotated reference sequences of the gene of interest obtained from http://plasmodb.org. The SNPs at *Pfk13* propeller region*, Pfmdr1* and *Pfcrt* genes were identified by comparing with reference 3D7 strains: PF3D7_1343700, PF3D7_0523000 and PF3D7_0709000, respectively.

### Data analysis

The count of samples with wild type and mutant alleles was used to generate the proportion of samples with SNPs. *Pfmdr1* haplotypes were constructed based on the permutations of SNPs at codons 86, 184, and 1246 [46]. The *Pfcrt* haplotype was constructed based on permutations of SNPs at codons 72–76 [44]. Samples with mixed infections (wild type and mutant nucleotides) were noted as such and counted as both wild and mutant haplotypes. For mixed infections, all possible *Pfmdr1* haplotype combinations (based on the observed SNPs) were reported, and the *Pfcrt* wild type (i.e., CVMNK) and most likely mutant haplotype (CVIDT and CVMDT) were reported, depending on the site of mutation (codons 74–76 and codons 75–76, respectively). Only samples from patients who experienced adequate clinical and parasitological response (ACPR) and paired samples (with successful amplification at both day-0 and day of failure) were included. Due to detection of both mutant and wild type codons in some of the samples, the totals do not sum to 100%.

The proportion of polymorphisms was compared between pre-treatment (day-0) samples obtained from children who experienced ACPR plus day-0 samples from children who were later re-infected *versus* children with recrudescent parasitaemia (day of failure samples). Additionally, the proportion of polymorphisms was compared between day-0 samples obtained from children who experienced ACPR plus day-0 samples from children who were later re-infected *versus* day of failure samples from all cases of recurrent infections (recrudescence and re-infection). Statistical significance of the association between any observed mutations (*Pfmdr1, Pfcrt* gene, or *Pfk13* propeller region polymorphisms) and the treatment outcome (day-0 samples obtained from children who experienced ACPR plus day-0 samples from children who were later re-infected *versus* recrudescent infections or day-0 samples obtained from children who experienced ACPR plus day-0 samples from children who were later re-infected *versus* recurrent infections) was tested using Fisher’s exact test. Statistical significance was defined by a p value of < 0.05 for all analyses, adjusted for multiple comparisons according to Bonferroni methods.

## Results

### Genotyping to characterize recurrent infections

Of the 110 recurrent infection samples genotyped at the *msp1*, *msp2*, and *glurp* alleles, 98 pairs of samples were successfully genotyped. A total of 24 recrudescent infections (16 and 8 in the AL and DP arms, respectively) and 74 re-infections (50 and 24 in the AL and DP arms, respectively) were identified.

### Proportion of *Pfk13* polymorphisms from day-0, recurrent and recrudescent infection samples

Sequencing of the *Pfk13* propeller region was successful for 98.1% day-0 samples, 80.0% recurrent infections, and 95.8% recrudescent samples (Table 1). No mutations were detected in the six codons that have been validated to correlate with artemisinin resistance. However, other non-synonymous mutations at the *Pfk13* propeller region which have not been associated with artemisinin resistance were detected; for day-0 samples, 0.6% had mutations at codon 522 (522**C)**, 1.9% had mutations at codon 578 (578**S**), and the remaining 99.4% samples were wild type (no or mixed mutation detected) at *Pfk13* propeller region. For recurrent infection samples, 2.3% had mutations at codon 578 (578**S**) and of the remaining samples, 98.9% were wild type for *Pfk13.* For recrudescent samples, 4.3% had mutations at codon 578 (578**S**) and of the remaining samples, 95.7% were wild type at *Pfk13* propeller region.

**Table 1:** Proportion of *Pfk13, Pfmdr1* and *Pfcrt* polymorphisms in day 0, recurrent and recrudescent infections

Polymorphism	Pre-treatment (day 0) samples	Recurrent infection samples	Recrudescent samples
*Pfk13*^a^			
Samples successfully sequenced	317/323 (98.1%)	88/110 (80.0%)	23/24 (95.8%)
Wild type (no or mixed mutations detected)	315/317 (99.4%)	87/88 (98.9%)	22/23 (95.7%)
522**C**	2/317 (0.6%)	0	0
578**S**	6/317 (1.9%)	2/88 (2.3%)	1/23 (4.3%)
*Pfmdr1*^a^			
Samples successfully sequenced	320/323 (99.1%)	95/110 (86.4%)	24/24 (100.0%)
N86	319/320 (99.7%)	94/95 (98.9%)	23/24 (95.8%)
86 **N/Y**	1/320 (0.3%)	0	0
86**Y**	0	1/95 (1.1%)	1/24 (4.2%)
Y184	129/320 (40.3%)	36/95 (37.9%)	8/24 (33.3%)
184**Y/F**	79/320 (24.7%)	16/95 (16.8%)	5/24 (20.8%)
184**F**	112/320 (35.0%)	43/95 (45.3%)	11/24 (45.8%)
D1246	290/320 (90.6%)	90/95 (94.7%)	22/24 (91.7%)
1246**D/Y**	19/320 (5.9%)	3/95 (3.2%)	2/24 (8.3%)
1246**Y**	11/320 (3.4%)	2/95 (2.1%)	0
NYD	203/320 (63.4%)	50/95 (52.6%)	13/24 (54.2%)
**YF**D	1/320 (0.3%)	1/95 (1.1%)	1/24 (4.2%)
N**F**D	185/320 (57.8%)	58/95 (61.1%)	15/24 (62.5%)
N**FY**	18/320 (5.6%)	2/95 (2.1%)	1/24 (4.2%)
NY**Y**	21/320 (6.6%)	5/95 (5.3%)	2/24 (8.3%)
**Y**YD	1/320 (0.3%)	0	0
**YFY**	0	0	0
**Y**Y**Y**	0	0	0
*Pfcrt*^a^			
Samples successfully sequenced	318/323 (98.5%)	95/110 (86.4%)	24/24 (100.0%)
C72	318/318 (100.0%)	95/95 (100.0%)	24/24 (100.0%)
72**S**	0	0	0
M74	314/318 (98.7%)	93/95 (97.9%)	23/24 (95.8%)
74 **M/I**	2/318 (0.6%)	0	0
74**I**	2/318 (0.6%)	2/95 (2.1%)	1/24 (4.2%)
N75	312/318 (98.1%)	93/95 (97.9%)	23/24 (95.8%)
75 **N/D**	4/318 (1.3%)	0	0
75**E**	2/318 (0.6%)	2/95 (2.1%)	1/24 (4.2%)
K76	312/318 (98.1%)	93/95 (97.9%)	23/24 (95.8%)
76 **K/T**	4/318 (1.3%)	0	0
76**T**	2/318 (0.6%)	2/95 (2.1%)	1/24 (4.2%)
CVMNK	316/318 (99.4%)	93/95 (97.9%)	23/24 (95.8%)
CV**IDT**^b^	2/318 (0.6%)	0	0
CVM**DT**^c^	2/318 (0.6%)	0	0
CV**IET**	2/318 (0.6%)	2/95 (2.1%)	1/24 (4.2%)

Bold letter denotes an encoded amino acid change; ^a^Totals may not sum due to mixed infections (samples with both wild and mutant codons) which were counted as both wild and mutant haplotypes). ^b^Two samples yielded the following findings for *Pfcrt*: C at site 72, M and I at site 74, N and D at site 75, K and T at site 76. These were considered mixed infections with CVMNK and CVIDT. ^c^Two samples yielded the following findings for *Pfcrt*: C at site 72, M at site 74, N and D at site 75, K and T at site 76. These were considered mixed infections with CVMNK and CVMDT

### Proportion of *Pfmdr1* polymorphisms in day-0, recurrent and recrudescent infections

The *Pfmdr1* gene was successfully sequenced for 99.1% of the day-0 samples, 86.4% of the recurrent infection samples, and 100% of the recrudescent samples (Table 1). A mutation at codon 86 (86**Y**) was identified in 0.3% of the day-0 samples, 1.1% of the recurrent infection samples, and 4.2% of the recrudescent samples. A mutation at codon 184 (184**F**) was identified in 59.7% of the day-0 samples, 62.1% of the recurrent infection samples, and 66.6% of recrudescent samples. A mutation at codon 1246 (1246**Y**) was identified in 9.3% of the tested day-0 samples, 5.3% of the recurrent infection samples, and 8.3% of the recrudescent samples. The proportion of NYD haplotypes was 63.4, 52.6, and 54.2% for the samples tested on day-0, recurrent infections and recrudescent infections, respectively. Mutations were not detected at codon 1034 (1034**C**) or 1042 (1042**D**) in any of the samples tested.

### Proportion of *Pfcrt* polymorphisms in day-0, recurrent and recrudescent samples

The *Pfcrt* gene was successfully sequenced for 98.5% of the day-0 samples, 86.4% of the recurrent infection samples, and 100% of the recrudescent samples (Table 1). A high proportion of samples harboured parasites with the *Pfcrt* chloroquine-sensitive genotype in codons 72–76 (CVMNK); 99.4, 97.9, and 95.8% in day-0, recurrent infection and recrudescent samples, respectively. A mutation at codon 74 (74**I**) was identified in 1.2% of day-0 samples, 2.1% of the recurrent infection samples, and 4.2% of the recrudescent samples. A mutation at codon 75 (75**D**/**E**) was identified in 1.9% of day-0 samples, 2.1% of the recurrent infection samples, and 4.2% of the recrudescent samples. A mutation at codon 76 (76**T**) was identified in 1.9% of day-0 samples, 2.1% of the recurrent infection samples, and 4.2% of the recrudescent samples. None of the samples tested had *Pfcrt* C72S mutations.

### *Pfpm2* gene copy number

All 8 pairs (day-0 and day of failure) of recrudescent samples from the DP arm analysed for *Pfpm2* gene copy number had a single gene copy.

### Association between *Pfk13*, *Pfmdr1* and *Pfcrt* polymorphisms and recurrent infections

Tables 2 and 3 show the proportions of *Pfk13*, *Pfmdr1* and *Pfcrt* polymorphisms in day-0 and day of failure samples in AL and DP treatment arm, respectively. In summary, there was no statistically significant association between any of the observed mutations (either SNPs or haplotypes) in the day-0 samples (ACPR plus day-0 samples from those who were later re-infected) and the treatment outcomes (day of failure due to re-infection and recrudescence or day of failure due to recrudescence only) in either the AL or DP treatment arms (Tables 2 and 3).

**Table 2:** Proportion of *Pfk13*, *Pfmdr1* and *Pfcrt* polymorphisms in day 0 and day of failure samples in Artemether−lumefantrine treatment arm

Molecular marker	Artemether−lumefantrine treatment arm				
	ACPR + Reinfection	Recrudescent-	*p*^a^	Recrudescent + Reinfection	*p*^*b*^
*Pfk13*^†^					
Wild type (no or mixed mutations detected)	129/130 (99.2%)	13/13 (100.0%)	**Ref**	58/58 (100.0%)	**Ref**
522**C**	1/130 (0.8%)	0	1	1/58 (1.7%)	0.53
578**S**	2/130 (1.5%)	0	1	0	1
*Pfmdr1*^†^					
N86	131/132 (99.2%)	15/15 (100.0%)	**Ref**	62/62 (100.0%)	**Ref**
86 **N/Y**	1/132 (0.8%)	0	1	0	1
86**Y**	0	0	1	0	1
Y184	53/132 (40.2%)	5/15 (33.3%)	**Ref**	23/62 (37.1%)	**Ref**
184**Y/F**	35/132 (26.5%)	3/15 (20.0%)	1	11/62 (17.7%)	0.31
184**F**	44/132 (33.3%)	7/15 (46.7%)	1	28/62 (45.2%)	0.90
D1246	116/132 (87.9%)	13/15 (86.7%)	**Ref**	57/62 (91.9%)	**Ref**
1246**D/Y**	10/132 (7.6%)	2/15 (13.3%)	1	3/62 (4.8%)	1
1246**Y**	6/132 (4.5%)	0	1	2/62 (3.2%)	1
NYD	85/132 (64.4%)	8/15 (53.3%)	**Ref**	32/62 (51.6%)	**Ref**
**YF**D	1/132 (0.8%)	0	1	0	1
N**F**D	76/132 (57.6%)	10/15 (66.7%)	1	39/62 (62.9%)	1
N**FY**	9/132 (6.8%)	1/15 (6.7%)	1	2/62 (3.2%)	1
NY**Y**	11/132 (8.3%)	2/15 (13.3%)	1	5/62 (8.1%)	1
**Y**YD	1/132 (0.8%)	0	1	0	1
**YFY**	0	0	1	0	1
**Y**Y**Y**	0	0	1	0	1
*Pfcrt*^†^					
C72	131/131 (100.0%)	15/15 (100%)	**Ref**	62/62 (100%)	**Ref**
72**S**	0	0	1	0	**1**
M74	128/131 (97.7%)	15/15 (100.0%)	**Ref**	62/62 (100.0%)	**Ref**
74 **M/I**	2/131 (1.5%)	0	1	0	1
74**I**	1/131 (0.8%)	0	1	0	1
N75	128/131 (97.7%)	15/15 (100.0%)	**Ref**	62/62 (100.0%)	**Ref**
75 **N/D**	2/131 (1.5%)	0	1	0	1
75**E**	1/131 (0.8%)	0	1	0	1
K76	128/131 (97.7%)	15/15 (100.0%)	**Ref**	62/62 (100.0%)	**Ref**
76 **K/T**	2/131 (1.5%)	0	1	0	1
76**T**	1/131 (0.8%)	0	1	0	1
CVMNK	130/131 (99.2%)	15/15 (100.0%)	**Ref**	62/62 (100.0%)	**Ref**
CVIDT^‡^	2/131 (1.5%)	0	1	0	1
CVMDT	0	0	1	0	1
CVIET	1/131 (0.8%)	0	1	0	1

ACPR: adequate clinical and parasitological response; ACPR + Reinfection: Samples collected pre-treatment (day 0) from participants without recurrent parasitaemia and from participants who were re-infected; Recrudescent: Samples collected on the day of failure from participants with recrudescent parasitaemia; Reinfection: Samples collected on the day of failure from participants who were re-infected; Ref: reference; ^a^Bonferroni adjusted statistical significance of difference in risk of treatment failure (recrudescence) determined by Fisher’s exact test; ^b^Bonferroni-adjusted statistical significance of difference in risk of recrudescence or reinfection determined by Fishers’s exact test. Bold letter denotes an encoded amino acid change; ^†^Totals may not sum due to mixed infections (samples with both wild and mutant codons) which were counted as both wild and mutant haplotypes); ^‡^Two samples yielded the following findings for *Pfcrt*: C at site 72, M and I at site 74, N and D at site 75, K and T at site 76. These were considered mixed infections with CVMNK and CVIDT

**Table 3:** Proportion of *Pfk13*, *Pfmdr1* and *Pfcrt* polymorphisms in day 0 and day of failure samples in Dihydroartemisinin−piperaquine treatment arm

Molecular marker	Dihydroartemisinin−piperaquine treatment arm				
	ACPR + Reinfection	Recrudescent	*p*^a^	Recrudescence + Reinfection	*p*^b^
*Pfk13*^†^					
Wild type (no or mixed mutations detected)	144/145 (99.3%)	7/8 (87.5%)	**Ref**	27/28 (96.4%)	**Ref**
522**C**	1/145 (0.7%)	0	1	0	1
578**S**	3/145 (2.1%)	1/8 (12.5%)	0.38	2/28 (7.1%)	0.38
*Pfmdr1*^†^					
N86	150/150 (100.0%)	8/9 (88.9%)	**Ref**	32/33 (97.0%)	**Ref**
86 **N/Y**	0	0	1	0	1
86**Y**	0	1/9 (11.1%)	0.18	1/33 (3.0%)	0.54
Y184	67/150 (44.7%)	3/9 (33.3%)	**Ref**	13/33 (39.4%)	**Ref**
184**Y/F**	28/150 (18.7%)	2/9 (22.2%)	1	5/33 (15.2%)	1
184**F**	55/150 (36.7%)	4/9 (44.4%)	0.36	15/33 (45.5%)	1
D1246	139/150 (92.7%)	9/9 (100%)	**Ref**	33/33 (100%)	**Ref**
1246**D/Y**	6/150 (4.0%)	0	1	0	1
1246**Y**	5/150 (3.3%)	0	1	0	1
NYD	93/150 (62.0%)	5/9 (55.6%)	**Ref**	18/33 (54.5%)	**Ref**
**YF**D	0	1/9 (11.1%)	0.48	1/33 (3.0%)	1
N**F**D	80/150 (53.3%)	5/9 (55.6%)	1	19/33 (57.6%)	1
N**FY**	7/150 (4.7%)	0	1	0	1
NY**Y**	7/150 (4.7%)	0	1	0	1
**Y**YD	0	0	1	0	1
**YFY**	0	0	1	0	1
**Y**Y**Y**	0	0	1	0	1
*Pfcrt*^†^					
C72	149/149 (100.0%)	9/9 (100.0%)	**Ref**	33/33 (100.0%)	**Ref**
72**S**	0	0	1	0	**1**
M74	148/149 (99.3%)	8/9 (88.9%)	**Ref**	31/33 (93.9%)	**Ref**
74 **M/I**	0	0	1	0	1
74**I**	1/149 (0.7%)	1/9 (11.1%)	0.33	2/33 (6.1%)	0.27
N75	146/149 (98.0%)	8/9 (88.9%)	**Ref**	31/33 (93.9%)	**Ref**
75 **N/D**	2/149 (1.3%)	0	1	0	1
75**E**	1/149 (0.7%)	1/9 (11.1%)	0.33	2/33 (6.1%)	0.27
K76	146/149 (99.3%)	8/9 (88.9%)	**Ref**	31/33 (93.9%)	**Ref**
76 **K/T**	2/149 (1.3%)	0	1	0	1
76**T**	1/149 (0.7%)	1/9 (11.1%)	0.33	2/33 (6.1%)	0.27
CVMNK	148/149 (99.3%)	8/9 (88.9%)	**Ref**	31/33 (93.9%)	**Ref**
CVIDT	0	0	1	0	1
CVMDT^‡‡^	2/149 (1.3%)	0	1	0	1
CVIET	1/149 (0.7%)	1/9 (11.1%)	0.33	2/33 (6.1%)	0.27

ACPR: adequate clinical and parasitological response; ACPR + Reinfection: Samples collected pre-treatment (day 0) from participants without recurrent parasitaemia and from participants who were re-infected; Recrudescent: Samples collected on the day of failure from participants with recrudescent parasitaemia; Reinfection: Samples collected on the day of failure from participants who were re-infected; Ref: reference; ^a^Bonferroni-adjusted statistical significance of difference in risk of treatment failure (recrudescence) determined by Fisher’s exact test. ^b^Bonferroni-adjusted statistical significance of difference in risk of recrudescence or reinfection determined by Fisher’s exact test. Bold letter denotes an encoded amino acid change; ^†^Totals may not sum due to mixed infections (samples with mixed infections were counted as both wild and mutant haplotypes); ^‡‡^Two samples yielded the following findings for *Pfcrt*: C at site 72, M at site 74, N and D at site 75, K and T at site 76. These were considered mixed infections with CVMNK and CVMDT

## Discussion

The use of ACT was recommended by the WHO in 2005 as a first-line treatment for falciparum malaria in all malaria-endemic countries [47]. This was due to the emergence and rapid spread of parasite resistance to previously recommended drugs, such as chloroquine and sulfadoxine-pyrimethamine [22]. ACT consists of an artemisinin component, which rapidly clears most parasites, and a longer acting partner drug, which eliminates remaining parasites and limits selection of drug resistance [47]. With the ongoing challenges related to the emergence of artemisinin resistance in multiple countries in Southeast Asia, including Cambodia, Thailand, Myanmar, and Laos [20, 27, 42, 43] and the threat of resistance emerging in sub-Saharan Africa [44], there is need for continued surveillance to monitor the efficacy of ACT and the genetic markers associated with anti-malarial drug resistance in malaria-endemic areas.

In this study, no *Pfk13* propeller region mutations previously validated to be correlated with artemisinin resistance were detected. These findings are consistent with previous studies conducted in western Kenya [29, 48] and coastal Kenya [49], which did not report any *Pfk13* propeller region mutations that have been associated with artemisinin resistance. The absence of these *Pfk13* mutations associated with artemisinin resistance in this study, as well as in other African countries [50], suggests that artemisinin resistance may not have emerged on the continent or spread from Southeast Asia.

Other non-synonymous mutations in the *Pfk13* propeller region, which have not been associated with resistance were detected; these were 522**C** and 578**S**. While a slightly higher proportion of the recurrent samples harboured the 578**S** mutant allele, at 2.3% compared with 1.9% in day-0 samples, these findings were seen without any impact on clinical resistance to artemisinin as observed by absence of parasites on day 3 [32]. Additionally, there was no statistically significant association between these observed mutations with either recrudescence or recurrent infections. The lack of association between the 578**S** polymorphism and a resistant phenotype is consistent with previous studies conducted in western Kenya and in four other African countries [27, 48] as well as multi-site studies in Southeast Asia and Africa that showed no association with drug resistance in patients treated with ACT [51]. Despite the lack of association of these mutations with a resistant phenotype, there is a need for further studies in a large population to assess whether *Pfk13* propeller region mutations are relevant in determining artemisinin resistance in parasite isolates in sub-Saharan Africa. It is possible that the actual SNP(s) that confer resistance differ from one geographic location to another depending on several factors, including parasite genetics, malaria transmission intensity, treatment seeking behaviour, and adherence to treatment guidelines. In multiple countries in Southeast Asia, where ACT resistance emerged, use of artemisinin monotherapy and sub-standard drugs is rampant and may partially explain why ACT resistance has emerged there but not in other areas [52].

The overall proportion of *Pfmdr1* 86**Y** mutant alleles in the current study was significantly lower (< 5%) than in a previous study conducted in western Kenya, which reported a prevalence of 69% in samples collected in 2010 [44]. The selection of *Pfmdr1* N86 wild type allele is likely due to the withdrawal of chloroquine and the widespread use of AL as the first-line anti-malarial treatment in Kenya, which could have promoted the selection of the wild-type sequences at this allele as observed in another study in western Kenya [27] and in other countries in Africa [20, 27, 53, 54]. The increasing levels of *Pfmdr1* N86 wild type allele could also suggest decreasing sensitivity to lumefantrine [24, 55] and artemisinin [7, 9, 11] as observed in previous studies. Additional studies are needed to assess the effect of N86 wild type allele on ACT susceptibility given the increasing proportion of samples harbouring this wild type allele over time in western Kenya.

The present study revealed a high proportion of the 184**F** mutation in the *Pfmdr1* gene with a proportion of 59.7, 62.1, and 66.6% for day-0, recurrent infections and recrudescent parasites, respectively. These results are consistent with a recent study conducted in western Kenya which reported a proportion of 65% after introduction of ACT [27]. The proportion of this mutant allele increased over time in comparison to a previous cross-sectional study in Siaya County, Kenya, which reported a prevalence of 23.3% in samples collected in 2010 [44]. The data suggest that the 184**F** mutant is possibly being selected for by ACT at the population level. However, there was no statistically significant association between the mutations with treatment failure (recrudescence) in both treatment arms in the current study. This is consistent with another study conducted in Senegal which did not find any association between *Pfmdr1* 184**F** mutants with susceptibility to various anti-malarial drugs in vitro [56]. A clinical trial assessing anti-malarial drug levels in patients has, however, associated *Pfmdr1* 184**F** mutants with reduced susceptibility to lumefantrine [14]. The role of this mutation as a marker of resistance in the current study could be unclear given that mutations were also present in the majority of successfully treated patients in both treatment arms. The discrepancy in conclusions from different studies regarding the role of 184**F** mutation in lumefantrine and other various anti-malarial drugs could be due to different study designs (i.e., in vitro studies, cross-sectional studies over time at population level, or clinical trials testing drug levels in patients), making it difficult to compare the data across different studies.

For codon 1246 of *Pfmdr1*, the proportion of mutant 1246**Y** alleles was reduced compared to previous studies in western Kenya, which reported prevalence of 40 and 16.5% [27, 44] compared to 9.3, 5.3, and 8.3% for day-0, recurrent infections and recrudescent samples, respectively in the current study. The decrease of the mutant alleles may be due to withdrawal of chloroquine and could also suggest decreased sensitivity to lumefantrine and artemisinin. In previous studies, the changes in lumefantrine sensitivity have been associated with polymorphisms in the *Pfmdr1* gene [24, 31]. For example, Tanzanian parasites having the *Pfmdr1* N**F**D (N86, 184**F**, D1246) haplotype were able to withstand lumefantrine blood concentrations 15-fold higher than parasites with the **Y**Y**Y** (86**Y**, Y184, 1246**Y**) haplotype [14]. In addition, in Uganda, AL was demonstrated to select for haplotypes with N86 in combination with 184**F** and D1246, or both [57].

In this study, a very high proportion (> 95%) of parasites harboured *Pfcrt* wild haplotype (CVMNK). This may be a result of the withdrawal of chloroquine and the introduction of ACT in 2006 in Kenya, which may have promoted the re-emergence of chloroquine-sensitive isolates. This is consistent with other studies conducted in Kenya [49, 58], Malawi [59], Côte d’Ivoire [60], and Tanzania [8], which have reported re-emergence of chloroquine-susceptible parasites following years of discontinuation of chloroquine use. A small proportion of parasites (< 5%) contained a mixed (N75**D**) or mutated (75**E**) nucleotide at codon 75, yielding haplotypes CV**IDT**, CVM**DT**, and CV**IET**. Prior literature suggests a stepwise mutation mechanism exists at codon 75, mutating from N75 to 75**D** to 75**E** [61]. Although CV**IET** is historically associated with chloroquine resistance [62], variable levels of chloroquine resistance have been noted in other *Pfcrt* isoforms, including a mixed mutation (N/**D**) at codon 75 [63]. Population-representative studies should be conducted with a larger number of samples from different regions to confirm the decrease of chloroquine-resistant parasites in Kenya. If the proportion of chloroquine-resistant parasites decreases at the national level to an undetectable level of *Pfcrt* mutants, a reintroduction of chloroquine in combination with other anti-malarial drugs for malaria treatment and prophylaxis may be considered in the context of ongoing molecular monitoring.

This study had several limitations. The study utilized samples obtained from children enrolled in a TES, which is not a population representative sample. Population representative studies are needed in order for the findings to be generalizable. Additionally, the sample size was limited to blood samples collected in the TES and was not powered to test for the association between parasite genotypes and treatment outcomes [46]. Because genotyping data are being collected as part of TESs conducted throughout the region and continent, metanalyses from similar studies across geographic areas may better assess the association between molecular markers and treatment outcome.

## Conclusion

The results indicate absence of mutations associated with parasite resistance to ACT in western Kenya. However, continued monitoring to evaluate the efficacy of anti-malarial drugs, particularly artemisinin-based combinations, is needed for providing timely evidence-based malaria treatment policies in western Kenya and other malaria-endemic regions.

## Data Availability

The data set used in this study is available and can be shared upon reasonable request to the corresponding author

## Abbreviations

ACPR: Adequate clinical and parasitological response
ACT: Artemisinin-based combination therapy
AL: Artemether−lumefantrine
CDC: Centers for Disease Control and Prevention
CGHR: Centre for Global Health Research
DP: Dihydroartemisinin–piperaquine
DF: Day of failure
GA: Georgia
*glurp*: Glutamate-rich protein
KEMRI: Kenya Medical Research Institute
*msp1*: Merozoite surface protein-1
*msp2*: Merozoite surface protein-2
PCR: Polymerase Chain Reaction
*Pfcrt*: *Plasmodium falciparum* chloroquine resistance transporter gene
*Pfk13*: *Plasmodium falciparum* kelch 13
*Pfmdr1*: *Plasmodium falciparum* multi-drug resistance protein 1
*Pfpm2*: *Plasmepsin 2*
SNP: Single nucleotide polymorphism
WHO: World Health Organization
